# Nuclear NF-κB p65 in Peripheral Blood Mononuclear Cells Correlates with Urinary MCP-1, RANTES and the Severity of Type 2 Diabetic Nephropathy

**DOI:** 10.1371/journal.pone.0099633

**Published:** 2014-06-17

**Authors:** Bin Yi, Xiaofang Hu, Hao Zhang, Jing Huang, Jishi Liu, Jing Hu, Wei Li, Lihua Huang

**Affiliations:** 1 Department of Nephrology, The Third Xiangya Hospital, Central South University, Changsha, Hunan, China; 2 Medical experimental center, The Third Xiangya Hospital, Central South University, Changsha, Hunan, China; TGen, United States of America

## Abstract

**Aims:**

To investigate if nuclear NF-κB p65 expression in *ex vivo* isolated peripheral blood mononuclear cells correlates with urinary MCP-1 or RANTES and the severity of type 2 diabetic nephropathy.

**Methods:**

According to their urinary albumin-to-creatinine ratio (uACR), 107 patients with type 2 diabetes (eGFR >60 ml/min) were divided into normal albuminuria group (DN0 group, 38 cases), microalbuminuria group (DN1 group, 38 cases), and macroalbuminuria group (DN2 group, 31 cases), compared with matched healthy normal control group (NC group, 30 cases). Nuclear NF-κB p65 protein expression levels in peripheral blood mononuclear cells were detected by western blotting. Real-time quantitative polymerase chain reaction was used to detect NF-κB p65 mRNA expression and ELISA assay was used to detect the levels of urinary MCP-1 and RANTES.

**Results:**

Nuclear NF-κB p65 protein and NF-κB p65 mRNA expression levels in peripheral blood mononuclear cells, urinary MCP-1/Cr and RANTES/Cr were all significantly higher in all diabetes groups as compared with NC group. In particular, the increase of nuclear NF-κB p65 protein and NF-κB p65 mRNA expressions, urinary MCP-1/Cr and RANTES/Cr all correlated with the severity of type 2 diabetic nephropathy as indicated by the increase in uACR. Pearson correlation analysis indicated that both urinary MCP-1/Cr and RANTES/Cr were positively correlated with nuclear NF-κB p65 protein or NF-κB p65 mRNA levels. Stepwise multiple regression analysis showed that nuclear NF-κB p65 protein or NF-κB p65 mRNA was an independent variable for urinary MCP-1/Cr, and MCP-1/Cr and RANTES/Cr were two independent variables for uACR.

**Conclusion:**

Our research demonstrates that nuclear NF-κB p65 protein and mRNA expressions in *ex vivo* isolated peripheral blood mononuclear cells well correlate with urinary MCP-1/Cr, RANTES/Cr and the severity of type 2 diabetic nephropathy.

## Introduction

Diabetic nephropathy (DN) is one of the many severe microvascular complications of diabetes. It’s reported that 20–40% of the patients with type 2 diabetes also develop DN [1]. In Western countries, DN is the first cause of end-stage renal disease (ESRD), and 44% of the DN patients eventually develop ESRD [2]. With China’s economic development, change in lifestyles and aging population, DN has become the second leading cause of ESRD in recent years, accounting for about 25% of the total ESRD patients, and the DN patient morbidity is continuously increasing [3]. The Third National Health and Nutrition Examination Survey (NHANES III) has analyzed the 10-year mortality rates in patients with diabetes and found that the standardized mortality rate in patients with DN was 31.1%, significantly higher than that in diabetic patients without DN, which was 11.5% [4].

It’s well known that the development of DN is related to metabolic disorders, genetic background, and environmental factors, but the exact underlying mechanism has not been fully understood. Recently, it has been suggested that DN is essentially a micro-inflammatory disease [5], and the role of micro-inflammation-mediated activation of intrinsic immune system in DN progression has attracted more and more attentions [6]. Monocyte chemoattractant protein (MCP) -1 and Regulated upon Activation, Normal T cell Expressed and Secreted (RANTES) are the key members of C-C chemokine subfamily. It has been shown that they can recruit mononuclear macrophages to glomerulus or tubulointerstitium. Macrophage infiltration is an important initial step in the inflammatory response of DN [7], and the degree of infiltration was positively correlated with the progression of DN tubulointerstitial fibrosis [8]. Previous studies have also shown that the promoters of both MCP-1 and RANTES genes contain binding sites for the transcription factor NF-κB [9], [10]. High glucose, advanced glycosylation end products, angiotensin II and other pathogenic factors for DN can all activate NF-κB pathway in mesangial cells and renal tubular epithelial cells, and then regulate the transcription and synthesis of MCP-1 and RANTES [10], [11]. However, little research has been done on the relationship between nuclear NF-κB expression in peripheral blood mononuclear cells, as a marker for activation of NF-κB signaling pathway, and MCP-1 and RANTES levels. In this study, we determined peripheral blood mononuclear cells nuclear NF-κB p65 protein and NF-κB p65 mRNA expressions, and urinary chemokine MCP-1 and RANTES levels in patients with type 2 diabetes and matched healthy controls. We further investigated the correlation between nuclear NF-κB p65 protein, NF-κB p65 mRNA levels and the severity of type 2 diabetic nephropathy.

## Materials and Methods

### Ethic Statement

The study was approved by the Ethics Committee of the Third Xiangya Hospital of Central South University. Written informed consent was obtained from each patient, who granted us permission to use the data obtained in subsequent studies.

### Study Subjects

107 patients with newly-diagnosed type 2 diabetes (eGFR >60 ml/min) hospitalized during January 2012–December 2012 in the Department of Nephrology and Endocrinology, Third Xiangya Hospital, Central South University, were included in this study. All patients are Chinese Han population with no kinship among them. Patients with the following conditions were excluded: patients with type 1 diabetes, secondary diabetes, concurrent diabetic ketoacidosis and hyperosmolar hyperglycemic state or under stress (such as severe infections, cardiovascular and cerebrovascular diseases, trauma and surgery), and those with chronic kidney disease, on vitamin D3, vitamin E, ACEI, AR-II blockers or any antihypertensive medication in the recent one month. According to 2007 KDOQI guidelines [12] on diabetic nephropathy staging criteria, all qualified patients were divided into normal albuminuria group (DN0 group, uACR <30 mg/g, 38 cases), microalbuminuria group (DN1 group, 30 mg/g ≤ uACR <300 mg/g, 38 cases) and macroalbuminuria group (DN2 group, uACR ≥300 mg/g, 31 cases) based on their spot urinary albumin/creatinine ratio (uACR). At the same time, age- and sex-matched healthy Han population without diabetes, chronic kidney disease, family history of these diseases, or cardiovascular and cerebrovascular diseases, were chosen as the normal control group (NC group, 30 cases).

### Study Methods

#### Clinical data

Sex, age, duration of disease, body mass index (BMI), waist-hip ratio, systolic blood pressure (SBP) and diastolic blood pressure (DBP) were collected for all study subjects. BMI = weight (kg)/height^2^ (m^2^).

#### Specimen collection

All subjects had more than 8 h overnight fasting before peripheral venous blood samples were drawn. Serum biochemical indices were measured and peripheral blood mononuclear cells were extracted. First morning urine samples were collected and centrifuged, with the supernatant stored at −80°C for further analysis.

#### Measurement of biochemical indices

Fasting blood glucose (FBG), serum albumin (Alb), serum creatinine (sCr), total cholesterol (TC) and triglycerides (TG) were measured using automatic biochemical analyzers. Glycosylated hemoglobin (HbA1c) was measured using high performance liquid chromatography, while urinary albumin (uALB) was measured using chemiluminescence methods. Urine creatinine (uCr) concentration was determined with biochemical assays. Estimated glomerular filtration rate (eGFR) was calculated using the MDRD formula, which is eGFR = 186× (Scr/88.4)^−1.154^×age^−0.203^× (0.742 for female subjects).

#### ELISA assay for measuring urinary MCP-1 and RANTES concentrations

The concentrations of MCP-1 and RANTES were determined using ELISA kit (R & D Systems, USA) according to the manufacturer’s instructions. To eliminate concentration errors, urinary MCP-1 and RANTES excretion rates were normalized by uCr and expressed as MCP-1/Cr and RANTES/Cr.

#### Real-time quantitative PCR for NF-κB p65 mRNA expression

PCR primers were designed using Oligo 6.0 software, and synthesized by Shanghai Sangon. NF-κB p65 primers: forward: 5′-ATCCCATCTTTGACAATCGTGC-3′, reverse: 5′-CTGGTCCCGTGAAATACACCTC-3′, and amplification product is 153 bp. GAPDH primers: forward: 5′-GCACCGTCAAGGCTGAGAAC-3′, reverse: 5′-TGGTGAAGACGCCAGTGGA-3′, and amplification product is 138 bp. Peripheral blood mononuclear cells were isolated using density gradient centrifugation and total RNA was extracted using TRIzol reagent (Invitrogen, USA). cDNA was synthesized using reverse transcription kit ReverTra Ace qPCR RT Kit (TOYOBO, JAPAN) according to the manufacturer’s instruction. Real-time PCR analysis was performed using the SYBR Green PCR Master Mix (TOYOBO, JAPAN) on an Applied Biosystems 7300 Sequence Detection System. PCR reactions were performed using the following cycle conditions: pre-denaturation at 95°C for two minutes, followed by denaturation at 95°C for 10 s, annealing at 58°C for 20 s, and extension at 72°C for 30 s at the end, with 40 cycles in total, followed by extension at 72°C for 5 min. The melting curve was used to confirm the specificity of the amplification products. The relative amounts of NF-κB and GAPDH mRNA were expressed as 2^−△CT^ (△CT = CT value of the target gene - CT value of internal control).

#### Western blotting for nuclear NF-κB p65 expression

For each group, nuclear NF-κB p65 protein expressions were evaluated in 12 randomly selected subjects from peripheral blood mononuclear cells isolated using density gradient centrifugation. Nuclear proteins were extracted using nucleoprotein kit (Merck, USA) according to the manufacturer’s instructions, and protein concentrations were measured using BCA protein assay kit (Pierce, USA). 30 µg of nuclear proteins from each sample were separated with SDS-PAGE gel electrophoresis. The resulted proteins were transferred to membrane, blocked with PBS containing 5% skim milk for 2 h, and then incubated with mouse anti-human NF-κB p65 antibody (1∶1000, CST, USA) or mouse anti-human proliferating cell nuclear antigen (PCNA) antibody (1∶4000, Santa Cruz, USA) at 4°C overnight. PCNA was used as internal control. The membrane was washed 3 times with TBST, incubated with HRP-conjugated goat anti-mouse IgG (1∶3000, Santa Cruz, USA) at room temperature for 2 h. Then the membrane was measured for the expression of protein using a chemiluminescent staining reagent kit and images were captured using Image Scanner. The gray value of individual protein band was measured using FluorChem8900 software, and the ratio of NF-κB p65 band to PCNA band was used to determine relative amount of nuclear NF-κB p65.

### Statistical Analysis

Database was built and data were analyzed using SPSS17.0 statistical software. Normally distributed quantitative data are expressed as mean ± standard deviation (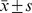). Numerical data are expressed as ratio. One-way ANOVA with post-hoc was applied to compare the value from each experimental group. Pearson correlation and stepwise multiple linear regression analysis were carried out to determine the correlations between clinical and testing data. With p<0.05, the difference was considered statistically significant.

## Results

### Analysis of Clinical Data and Biochemical Indices

As shown in Table 1, there was no statistical difference in age, gender, BMI, waist-hip ratio, DBP, or TC, among experimental groups or between experimental groups and the control group. There was no statistical difference in the disease duration, FBG, HbA1c, serum Alb or TG among all diabetes groups. However, FBG and TG levels were significantly higher in all diabetes groups than the NC group, and serum albumin concentration was significantly lower in diabetes groups than the NC group. The SBP in all diabetes groups was significantly higher compared to that in the NC group, and the SBP in DN2 group was significantly higher than DN0 and DN1 groups. The eGFR level was significantly lower in DN2 group compared to NC, DN0 or DN1 groups, but there was no significant difference between NC, DN0 and DN1 groups.

**Table 1:** Comparison of clinical data (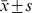).

Subjects	NC Group	DN0 Group	DN1Group	DN2 Group
	(n = 30)	(n = 38)	(n = 38)	(n = 31)
Age (y)	56.57±8.78	56.26±11.38	55.87±11.87	59.52±14.14
Sex (M/F)	16/14	20/18	21/17	17/14
BMI (kg/m^2^)	23.35±1.73	24.60±3.05	24.63±2.75	23.65±2.89
Waist-hip ratio	0.95±0.05	0.96±0.06	0.96±0.05	0.96±0.05
Duration of disease (y)	-	6.79±6.11	7.74±7.01	8.00±5.17
SBP (mmHg)	125.83±10.45	133.08±17.83*	139.21±16.13*	150.61±18.87*^#△^
DBP (mmHg)	77.50±7.78	78.11±11.84	82.08±10.83	83.68±9.41
FBG (mmol/l)	4.99±0.49	7.95±2.40*	7.83±2.77*	7.91±2.57*
HbA1c (%)	-	8.84±2.17	9.55±2.51	8.77±1.99
ALB (g/L)	43.65±4.45	40.58±4.80*	40.61±3.46*	37.97±6.67*
TG (mmol/l)	1.39±0.33	1.68±0.71*	1.70±0.82*	2.16±1.21*
TC (mmol/l)	4.56±0.89	5.03±1.16	4.78±1.21	5.12±1.22
uACR (ug/mg)	9.84±3.37	19.92±7.58*	83.31±29.92*^#^	912.44±432.12*^#△^
eGFR (ml/min)	115.34±21.36	109.15±24.39	111.27±20.05	85.6745±24.39*^#△^

Note: Results are expressed as mean ± SD and ratio. Compared with NC group *: P<0.05; compared with DN0 group #: P<0.05; compared with DN1 group △: P<0.05; - no data.

BMI: Body mass index. SBP: Systolic blood pressure. DBP: Diastolic blood pressure. FBG: Fasting blood glucose. Alb: serum albumin. HbA1c: Hemoglobin A1c. TC: total cholesterol. TG: triglyceride. uACR: Urinary albumin creatinine ratio. eGFR: estimated glomerular filtration rate (According to MDRD formula).

### Expression of Peripheral Blood Mononuclear Cell Nuclear NF-κB P65

**Figure 1:**
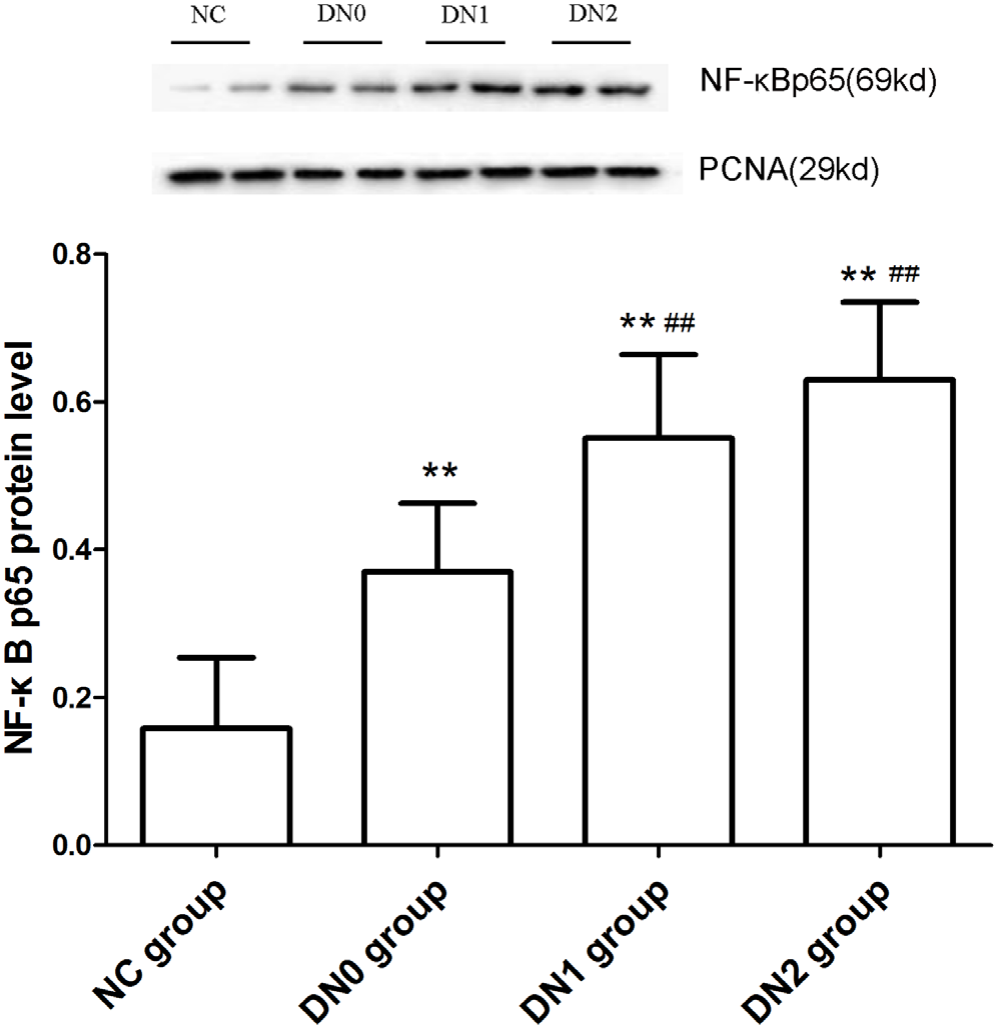
Nuclear NF-κB p65 protein level in peripheral blood mononuclear cells determined by western blotting. Note: Compared with NC group **: P<0.01; compared with DN0 group ##: P<0.01.

As shown in the western blotting results (Figure 1), the nuclear NF-κB p65 expression of peripheral blood mononuclear cell in NC group was the lowest, with gradual increase in DN0, DN1 and DN2 groups, respectively, and with DN2 group being the highest. All differences were statistically significant compared with NC group. Notably, NF-κB p65 protein expression was significantly higher in DN2 and DN1 groups than that in DN0 group (Figure 1).

**Figure 2:**
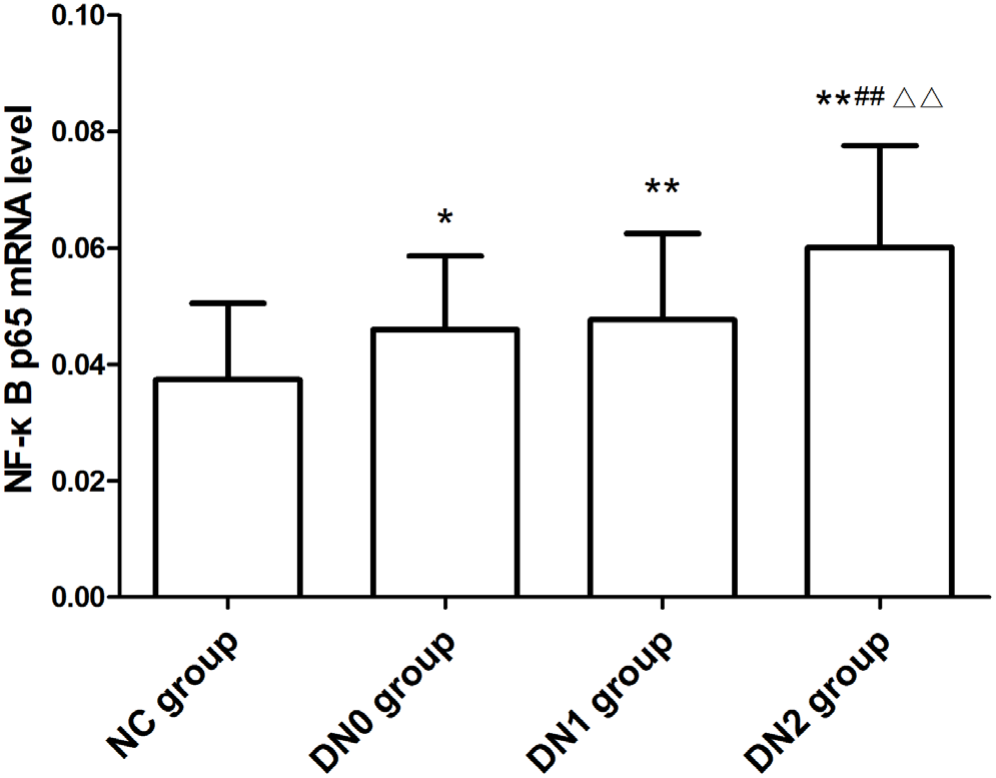
Nuclear NF-κB p65 mRNA level in peripheral blood mononuclear cells determined by real-time quantitative PCR. Note: Compared with NC group *: P<0.05, **: P<0.01; compared with DN0 group ##: P<0.01; compared with DN1 group △△: P<0.01.

In addition, we also measured NF-κB p65 mRNA levels in peripheral blood mononuclear cells of all subjects. Real-time quantitative RT-PCR showed that the expression of NF-κB p65 mRNA was significantly increased in DN2, DN1 or DN0 groups when compared to NC group. Especially, DN2 group had higher NF-κB p65 mRNA expression than DN1 or DN0 groups (Figure 2).

### Excretion Rates of Urinary MCP-1 and RANTES

**Figure 3:**
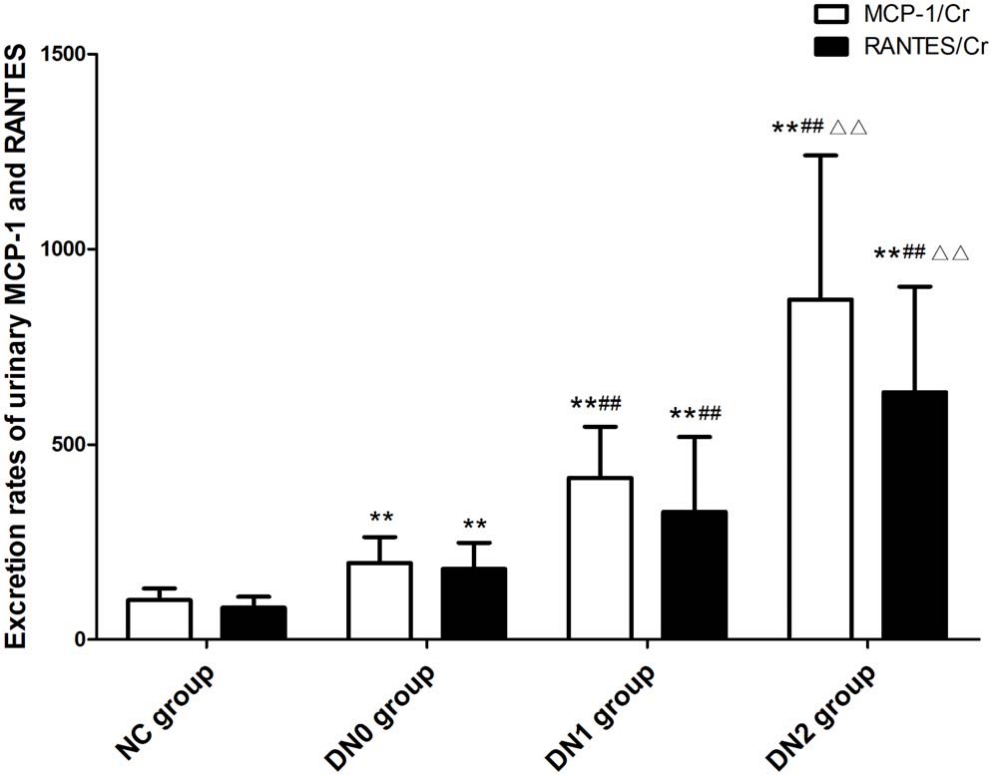
Excretion rates of urinary MCP-1 and RANTES. Note: Compared with NC group **: P<0.01; compared with DN0 group ##: P<0.01; compared with DN1 group △△: P<0.01.

Urinary MCP-1/Cr in NC, DN0, DN1 and DN2 groups were 101.14±29.47 pg/mgCr, 196.07±66.27 pg/mgCr, 414.57±130.93 pg/mgCr and 870.75±369.76 pg/mgCr, respectively. There were significant differences among all groups. Urinary RANTES/Cr in NC, DN0, DN1 and DN2 groups were 81.39±27.89 pg/mgCr, 180.63±67.55 pg/mg, 327.47±191.88 pg/mgCr and 632.74±270.84 pg/mgCr, respectively. There were significant differences among all groups (Figure 3).

### Correlation Analysis between Nuclear NF-κB P65 Protein and NF-κB P65 mRNA Expressions in Peripheral Blood Mononuclear Cells, Urinary MCP-1, and RANTES Excretion Rates in Type 2 Diabetes Patients

**Figure 4:**
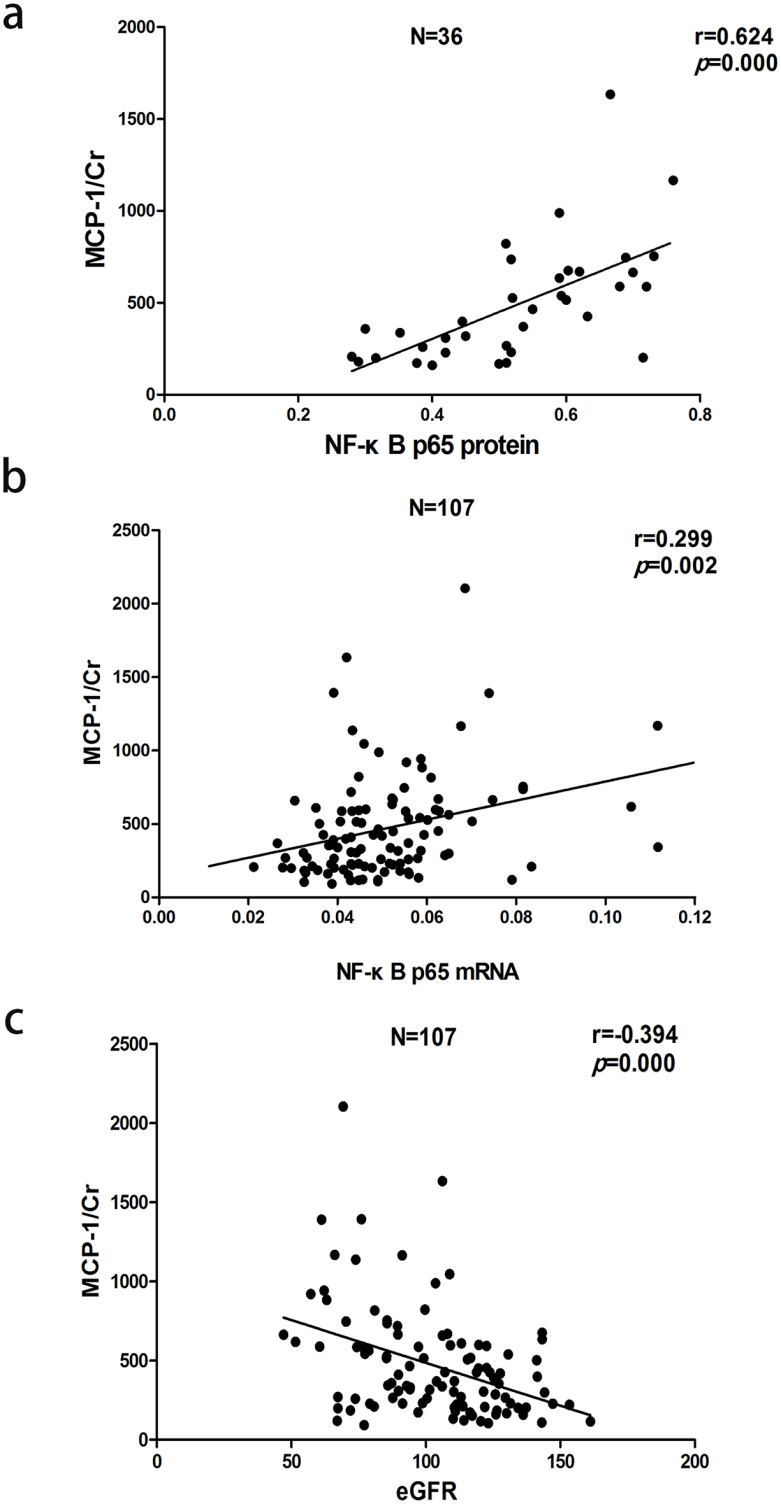
Correlation between urinary MCP-1 excretion rate and (a) nuclear NF-κB p65 protein, (b) NF-κB p65 mRNA or (c) eGFR.

Pearson correlation analysis indicated that urinary MCP-1/Cr was positively correlated with nuclear NF-κB p65 protein (r = 0.624, P<0.01), NF-κB p65 mRNA (r = 0.299, P<0.01) and SBP (r = 0.324, P<0.01), negatively correlated with eGFR (r = −0.394, P<0.01). Further stepwise multiple regression analysis indicated that nuclear NF-κB p65 protein (β = 0.450, P = 0.003) and NF-κB p65 mRNA (β = 0.182, P = 0.049) were both the independent variables of urinary MCP-1/Cr (Figure 4).

**Figure 5:**
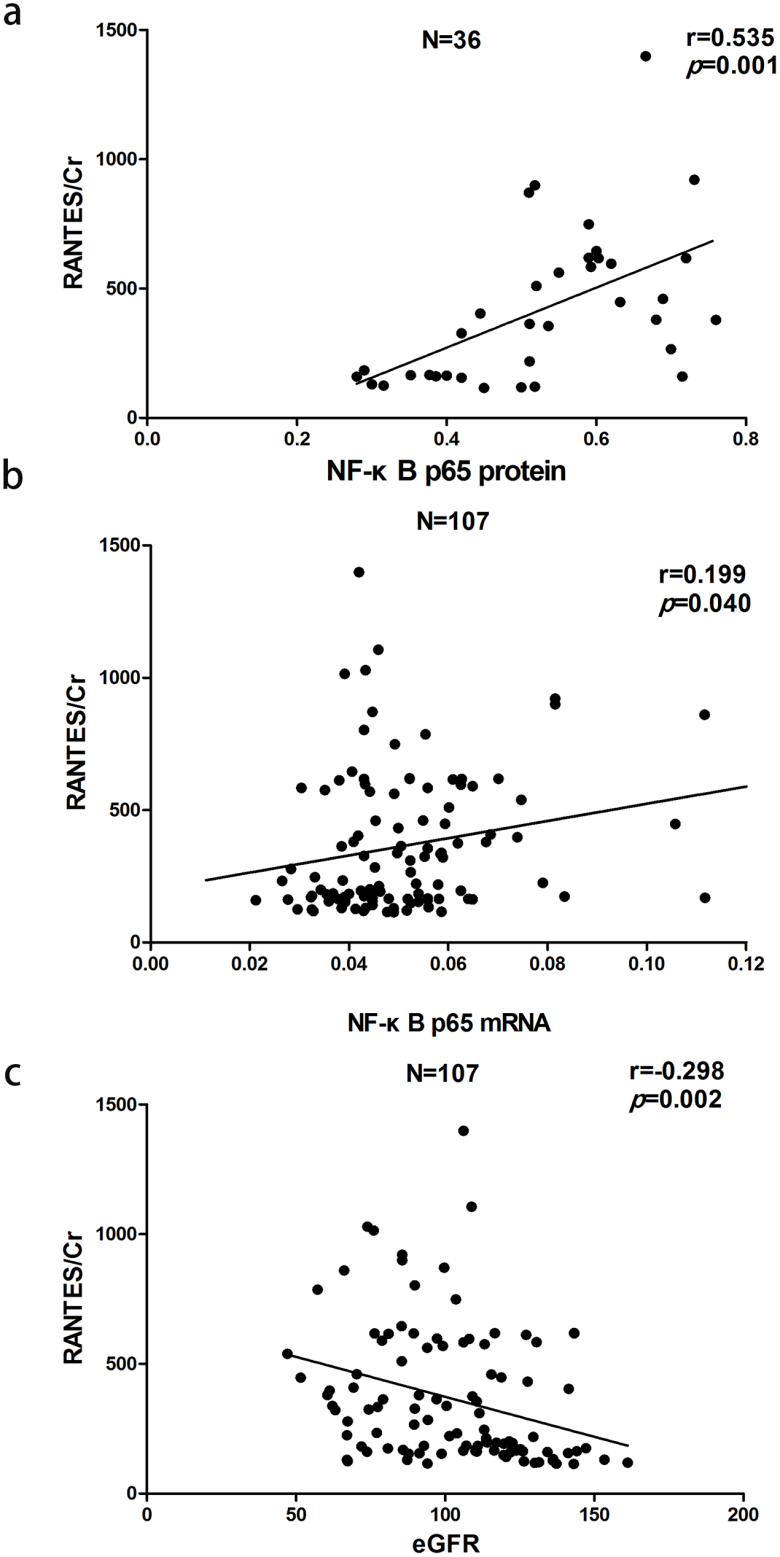
Correlation between urinary RANTES excretion rate and (a) nuclear NF-κB p65 protein, (b) NF-κB p65 mRNA or (c) eGFR.

Urinary RANTES/Cr was positively correlated with nuclear NF-κB p65 protein (r = 0.535, P<0.01), NF-κB p65 mRNA (r = 0.199, P<0.05), SBP (r = 0.261, P<0.01) and eGFR (r = −0.298, P<0.01). Stepwise multiple regression analysis indicated that nuclear NF-κB p65 protein (β = 0.493,P = 0.001), but not NF-κB p65 mRNA, was an independent variable of urinary RANTES/Cr (Figure 5).

### Correlation Analysis between uACR, Urinary Chemokines and Peripheral Blood Mononuclear Cell NF-κB P65 in Type 2 Diabetic Patients

**Figure 6:**
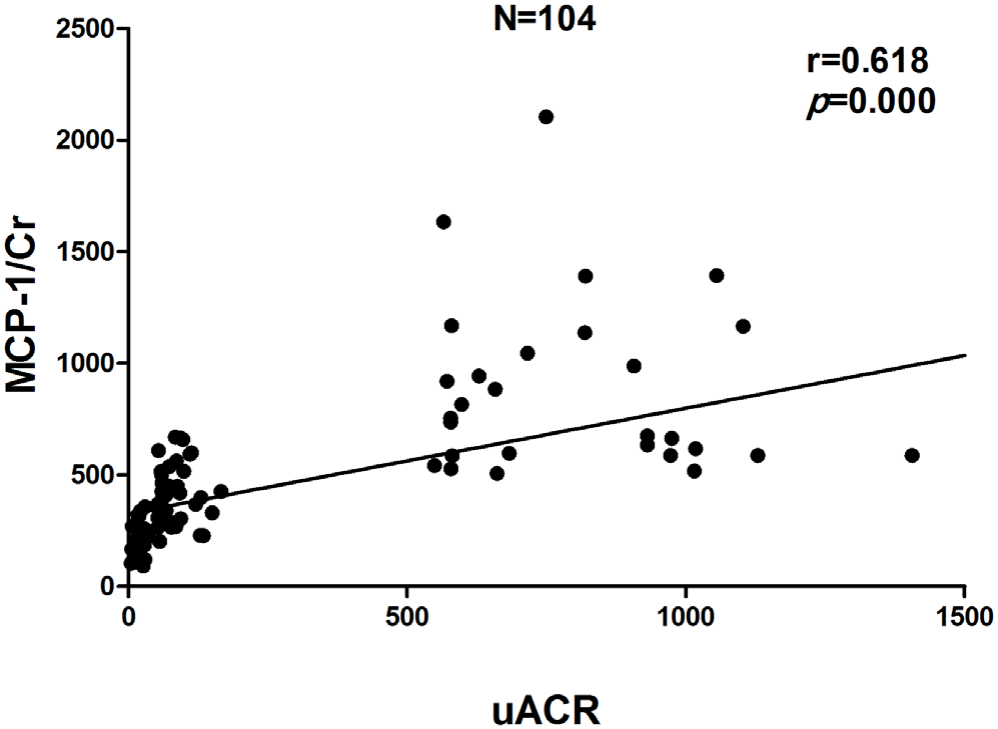
Correlation between urinary MCP-1 excretion rate and uACR.

**Figure 7:**
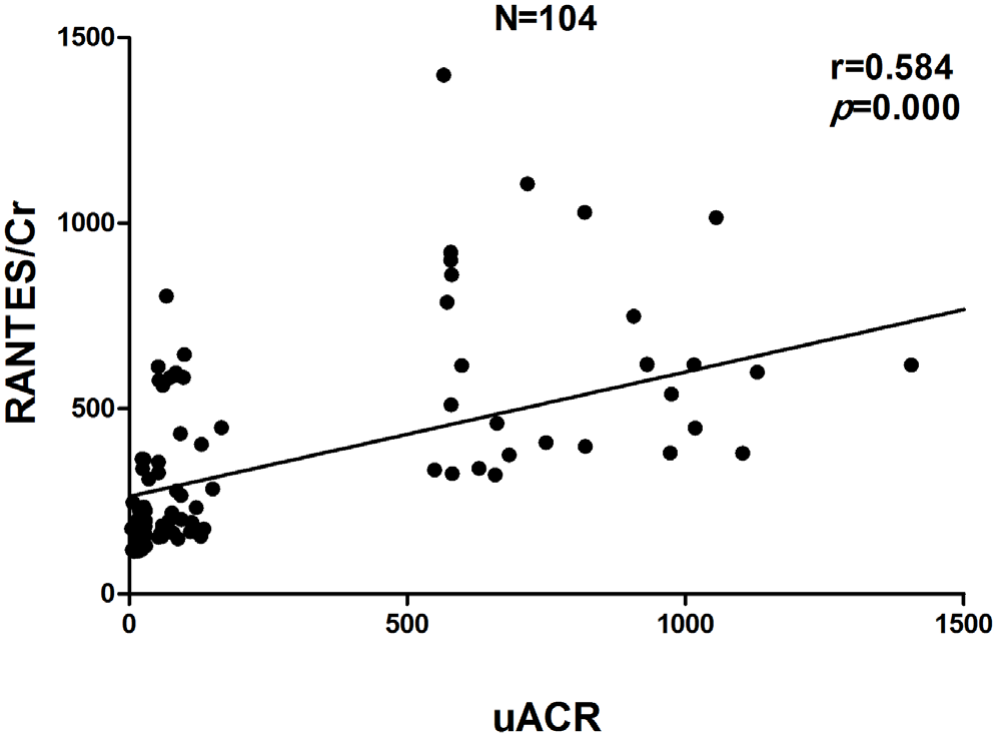
Correlation between urinary RANTES excretion rate and uACR.

**Figure 8:**
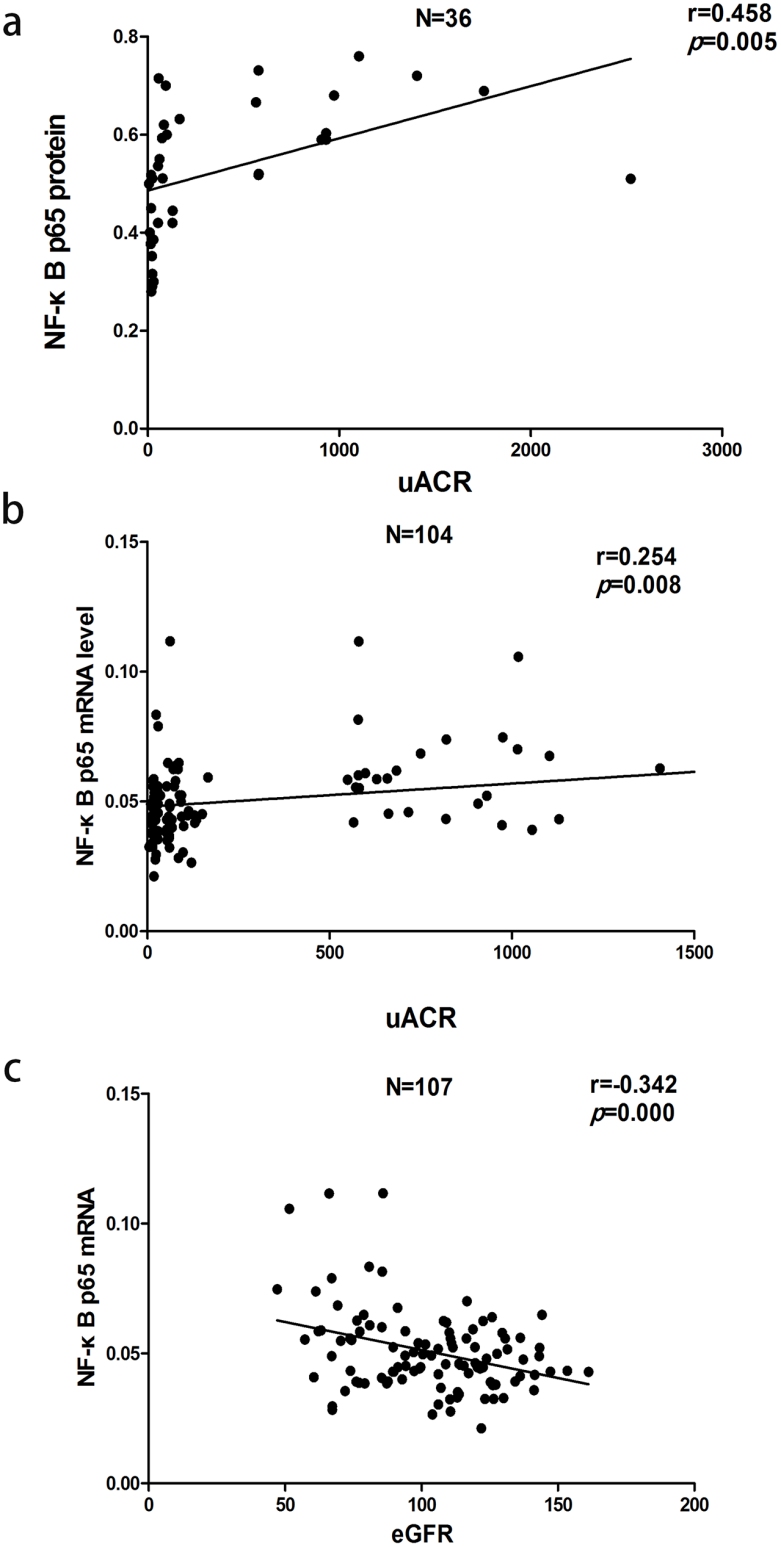
Correlation between peripheral blood mononuclear cell (a) nuclear NF-κB p65 protein and uACR (b) NF-κB p65 mRNA and uACR, or (c) NF-κB p65 mRNA and eGFR.

Pearson correlation analysis indicated that uACR was positively correlated with MCP-1/Cr (r = 0.618, P<0.01), RANTES/Cr (r = 0.584, P<0.01), SBP (r = 0.405, P<0.01), nuclear NF-κB p65 protein (r = 0.458,P = 0.005) and NF-κB p65 mRNA (r = 0.254, P = 0.008), and negatively correlated with eGFR (r = −0.362, P<0.01). We analyzed the correlation between NF-κB and eGFR and found that NF-κB p65 mRNA (r = −0.342, P<0.01), but not NF-κB p65 protein (r = −0.080, P>0.05), was negatively correlated with eGFR. Further stepwise multiple regression analysis showed that both urinary MCP-1/Cr (β = 0.324, P = 0.001) and RANTES/Cr (β = 0.286, P = 0.003) were independent variables for uACR. There was no independent correlation between nuclear NF-κB p65 protein, or NF-κB p65 mRNA and uACR (Figures 6–8).

## Discussion

Many recent studies have indicated that DN is essentially a micro-inflammatory disease, where the interactions between various pro-inflammatory cytokines and chemokines cause inflammatory cascade, leading to pathological changes in the structure and function in DN patients [6]. NF-κB is the common transcriptional regulatory protein for many inflammatory factors, and has played an important role in the regulation of chemokine MCP-1 and RANTES-induced DN macrophage infiltration [13].

In this study, we have found that both the nuclear protein and mRNA levels of peripheral blood mononuclear cell NF-κB p65 were higher in all diabetes groups than the control group, and were gradually elevated with the progression of type 2 diabetic nephropathy as indicated by the increase of uACR and proceeded to a peak expression in the macroalbuminuria group. As shown by Pearson correlation analysis, both nuclear NF-κB p65 protein and NF-κB p65 mRNA were positively correlated with uACR in type 2 diabetes patients. Chen et al. [14] have shown that the expression of NF-κB was significantly increased in the renal tissues of STZ-induced DN rats, and was positively correlated with 24-hour urinary protein concentration. The number of NF-κB-positive cells was also closely related to the mononuclear macrophage infiltration and pathological changes in renal structure and function. Hofmann et al. [15] examined the activity of peripheral blood mononuclear cell NF-κB signaling in 33 diabetic patients and found that NF-κB activity was significantly increased in DN patients compared to those without renal impairment, and was closely related to the degree of albuminuria. In consistent with these previous findings, our study showed that the nuclear expression of transcriptional regulatory protein NF-κB, as a marker for activation of NF-κB signaling pathway, correlates with the severity of DN in patients with type 2 diabetes. Additionally, NF-κB inhibitor Celastrol significantly reduced fasting blood glucose and glycosylated hemoglobin, as well as serum creatinine and urinary albumin excretion rate in db/db diabetic mice [16], suggesting that inhibition of NF-κB activation might play an indispensable role in preventing the progression of DN. Our results demonstrate that NF-κB p65 mRNA, but not nuclear NF-κB p65 protein, is negatively correlated with eGFR, which is one of the best indicators of renal functions. One possible reason is that all patients enrolled in our current study are with stage 1 or 2 chronic kidney disease (eGFR >60 ml/min), and the exclusion of stage 3 to 5 chronic kidney disease patients might contribute to the limitation of our observations. Another potential explanation might be that the increase of uACR identifies the early stage of diabetic nephropathy, while the reduction of eGFR is closed correlated with glomerular sclerosis or tubulointerstitial fibrosis which happens as results of systematic inflammation in the late stage of diabetic nephropathy. Since renal function deterioration directly follows the emergence of macroalbuminuria in DN patients, a prospective study including all five stages of chronic kidney diseases will be our next clinical approach to further clarify the relationship between eGFR and NF-κB p65 in peripheral blood mononuclear cells.

Our studies have also shown that urinary MCP-1/Cr and RANTES/Cr were higher in all groups with type 2 diabetes compared with normal control group, and gradually increased as uACR elevated. There were significant differences between macroalbuminuria and microalbuminuria groups, and similarly between microalbuminuria and normal albuminuria groups. Tam et al. [17] have followed 40 diabetes patients for six years and found that urinary MCP-1/Cr was significantly higher during macroalbuminuria period compared to normal albuminuria and microalbuminuria periods for each individual. Consistent with previous reports by Wang et al. [18] and Bondar et al. [19], our study has shown that urinary MCP-1 and RANTES excretion rates have significantly increased in DN patients, and the rates were higher in macroalbumineria group than in microalbuminuria and normal albuminuria groups. Pearson correlation analysis indicated that urinary MCP-1/Cr and RANTES/Cr were positively correlated with uACR in patients with type 2 diabetes, with r value at 0.618 and 0.584, respectively. Further multiple regression analysis indicated that MCP-1/Cr and RANTES/Cr were independently correlated with uACR. In addition, we also find that MCP-1/Cr and RANTES/Cr were negatively correlated with eGFR. All these results suggest that chemokine MCP-1 and RANTES might directly correlate with the severity of type 2 DN, and are the important indicators of the progression of albuminuria.

The promoters of MCP-1 and RANTES genes both contain binding sites for transcription factor NF-κB [9], [10]. In vivo studies have shown that inhibition of NF-κB activation reduces T cell and macrophage infiltration and RANTES expression in mouse kidneys with unilateral ureteral obstruction [20]. Mezzano et al. [13] have found that activation of NF-κB in renal tubular epithelial cells was highly correlated with expression of renal interstitial MCP-1 and RANTES in patients with type 2 DN, suggesting that activation of NF-κB pathway can induce macrophage infiltration and promote renal interstitial fibrosis by activating MCP-1 and RANTES.

It has been shown that ACEI or aldosterone inhibitors can improve DN by inhibiting the RAAS system to reduce urinary MCP-1 levels [21], [22]. The possible underlying mechanism is that angiotensin II activates NF-κB pathway to regulate transcription and synthesis of MCP-1 and RANTES [10], [11]. In our studies, we have shown by Pearson correlation analysis that peripheral blood mononuclear cell nuclear NF-κB p65 protein or NF-κB p65 mRNA was positively correlated with urinary MCP-1/Cr and RANTES/Cr in patients with type 2 diabetes. Further multiple linear regression analysis indicated that nuclear NF-κB p65 protein or NF-κB p65 mRNA expression was independently correlated with urinary MCP-1/Cr. These results indicated that NF-κB activation could promote activation of chemokine MCP-1 and subsequently increase urinary MCP-1 excretion in patients with type 2 diabetes. On the other hand, nuclear NF-κB p65 protein, but not NF-κB p65mRNA, was an independent variable of urinary RANTES/Cr, indicates that nuclear NF-κB p65 protein might be a better marker reflecting NF-κB activation and subsequent activation of RANTES.

In conclusion, type 2 DN is an inflammatory disease and nuclear NF-κB p65 expression and NF-κB p65 mRNA in *ex vivo* isolated peripheral blood mononuclear cells well correlates with urinary MCP-1/Cr, RANTES/Cr and the severity of type 2 diabetic nephropathy, suggesting that systemic inflammation is involved in the pathogenesis of type 2 DN.
